# Shared Gene Expression and Immune Pathway Changes Associated with Progression from Nevi to Melanoma

**DOI:** 10.3390/cancers14010003

**Published:** 2021-12-21

**Authors:** Elizabeth S. Borden, Anngela C. Adams, Kenneth H. Buetow, Melissa A. Wilson, Julie E. Bauman, Clara Curiel-Lewandrowski, H.-H. Sherry Chow, Bonnie J. LaFleur, Karen Taraszka Hastings

**Affiliations:** 1 Department of Basic Medical Sciences, University of Arizona College of Medicine Phoenix, Phoenix, AZ 85004, USA; knodele@email.arizona.edu (E.S.B.); anngelaa@arizona.edu (A.C.A.); 2 Phoenix Veterans Affairs Health Care System, Phoenix, AZ 85012, USA; 3 School of Life Sciences, Arizona State University, Tempe, AZ 85281, USA; kenneth.buetow@asu.edu (K.H.B.); mwilsons@asu.edu (M.A.W.); 4 Center for Evolution and Medicine, Arizona State University, Tempe, AZ 85281, USA; 5 Department of Medicine, University of Arizona College of Medicine Tucson, Tucson, AZ 85724, USA; jebauman@arizona.edu (J.E.B.); ccuriel@arizona.edu (C.C.-L.); schow@arizona.edu (H.-H.S.C.); 6 University of Arizona Cancer Center, University of Arizona, Tucson, AZ 85724, USA; 7 BIO5 Institute, University of Arizona, Tucson, AZ 85724, USA; blafleur@arizona.edu

**Keywords:** melanoma, dysplastic nevi, molecular biomarkers

## Abstract

**Simple Summary:**

Melanoma is a deadly skin cancer, and the incidence of melanoma is rising. Chemoprevention, using small molecule drugs to prevent the development of cancer, is a key strategy that could reduce the burden of melanoma on society. The long-term goal of our study is to develop a gene signature biomarker of progression from nevi to melanoma. We found that a small number of genes can distinguish nevi from melanoma and identified shared genes and immune-related pathways that are associated with progression from nevi to melanoma across independent datasets. This study demonstrates (1) a novel approach to aid melanoma chemoprevention trials by using a gene signature as a surrogate endpoint and (2) the feasibility of determining a gene signature biomarker of melanoma progression.

**Abstract:**

There is a need to identify molecular biomarkers of melanoma progression to assist the development of chemoprevention strategies to lower melanoma incidence. Using datasets containing gene expression for dysplastic nevi and melanoma or melanoma arising in a nevus, we performed differential gene expression analysis and regularized regression models to identify genes and pathways that were associated with progression from nevi to melanoma. A small number of genes distinguished nevi from melanoma. Differential expression of seven genes was identified between nevi and melanoma in three independent datasets. C1QB, CXCL9, CXCL10, DFNA5 (GSDME), FCGR1B, and PRAME were increased in melanoma, and SCGB1D2 was decreased in melanoma, compared to dysplastic nevi or nevi that progressed to melanoma. Further supporting an association with melanomagenesis, these genes demonstrated a linear change in expression from benign nevi to dysplastic nevi to radial growth phase melanoma to vertical growth phase melanoma. The genes associated with melanoma progression showed significant enrichment of multiple pathways related to the immune system. This study demonstrates (1) a novel application of bioinformatic approaches to aid clinical trials of melanoma chemoprevention and (2) the feasibility of determining a gene signature biomarker of melanomagenesis.

## 1. Introduction

Melanoma is the sixth most common cancer in both men and women in the US. The vast majority of melanoma cases are cutaneous malignant melanoma (CMM). Solar ultraviolet radiation (UVR) exposure remains the major environmental risk factor for developing CMM. Despite public health initiatives that have promoted sun protection, the incidence of CMM continues to increase, with much of the increased incidence driven by thin CMM [1,2]. According to recent predictions from the American Cancer Society, an estimated 106,110 new cases of invasive melanoma and 101,280 cases of melanoma in situ will be diagnosed in the US in 2021 [3]. Despite recent advances in targeted therapies as well as immunotherapies, 7180 melanoma-related deaths are estimated for 2021 [3]. The increasing incidence of CMM, poor prognosis, and societal cost in treating advanced stages support the investigation of novel approaches to prevention such as chemoprevention.

To date, limited early phase clinical trials have been conducted to evaluate potential chemopreventive agents for melanoma prevention (reviewed by [4]). In these trials, the chemopreventive effects are often evaluated in individuals with clinically atypical or histopathologically dysplastic nevi (DN), because several lines of evidence support the notion that DN are markers of increased risk for CMM and could be potential precursors of CMM. The presence of one or more atypical or DN, or 25 or more benign nevi (BN), are clinical risk factors for the development of CMM [5]. About 26% of all invasive CMM contain histologic features of nevus [6]. In this study, we term the nevus portion of a CMM arising in the context of a nevus as progressing nevi (PN) for nevi known to progress to melanoma. These findings support the relevance of investigating DN and PN lesions, in comparison to CMM, to identify genes associated with progression towards CMM.

Early phase clinical trials have included clinical and histologic changes of DN as early efficacy biomarkers, but failed to demonstrate any changes in these endpoints [7,8,9]. However, it is not known whether the clinical and histologic features of DN can be modulated with the limited intervention duration in the setting of early phase trials. In addition, the few selected molecular biomarkers included in the prior trials had only putative associations with the degree of dysplasia [7,9]. Thus, developing molecular biomarkers of the progression toward melanoma to assess the early efficacy of a candidate agent represents an important area of research in the clinical development of chemopreventive agents for melanoma prevention.

In this study, we sought to determine the changes in gene expression associated with melanoma progression, using three publicly available datasets with gene expression data for DN, PN, and CMM. Differential gene expression analysis and regularized regression models identified genes that distinguished nevi (both DN and PN) from CMM across multiple datasets. The genes identified in this study have known expression and functions consistent with a role in melanoma progression, supporting the feasibility of this approach to create a gene signature biomarker of melanoma progression to assist clinical trials of putative chemopreventive agents.

## 2. Materials and Methods

### 2.1. Data Acquisition and Processing

Three publicly available datasets containing gene expression data, including DN or PN and CMM, were obtained using the data accession numbers presented in Table 1, briefly the Krueger dataset, the Scatolini dataset, and the Bastian dataset. These datasets were identified by searching the dbGaP and GEO databases for datasets including both CMM and DN or PN with expression data from microarrays or RNA sequencing (RNAseq). In the Krueger dataset, BN included congenital, compound, intradermal, and junctional nevi; DN included compound nevi with moderate or severe atypia; and CMM included invasive melanoma with a Breslow depth between 0.4 and 6.7 mm [10,11]. However, annotations were not provided for the subset of lesions with publicly available data. The samples were selected from frozen sections of the tumor. In the Scatolini dataset, the BN characteristics were not specified, the DN included both low- and high-grade atypia, and the CMM samples included both melanoma in situ and invasive melanoma. Scatolini further divided their CMM into radial growth phase melanoma (RGM), defined as Clark level I and II, and vertical growth phase melanoma (VGM), defined as Clark level III, IV, and V. For BN, DN and RGM, the area representative of the lesion was selected from the fresh, excisional biopsy, and for VGM, the specimen was limited to the dermal portion [12]. In the Bastian dataset, formalin-fixed paraffin-embedded samples of CMM arising in the context of a nevus were manually microdissected with a scalpel under a dissecting microscope. We refer to the nevus portion as PN and invasive melanoma portion as CMM [13,14].

The two microarray datasets (Krueger and Scatolini) were obtained as pre-processed expression values. Probe names for both datasets were converted to gene names using the DAVID Web Services to ensure that the annotations for each dataset were consistent with the official gene names and up-to-date gene mappings for each probe [15]. Only probes with an existing, gene-level annotation in DAVID were considered in the analyses. Multiple probes for the same gene were averaged to obtain gene-level data. These analyses were repeated with a probe-level approach and resulted in similar sets of differentially expressed genes.

Krueger and colleagues performed quality control, assessed for spatial artifacts, processed the data, and provided the log_2_ GCRMA expression values [11], where probe intensities were normalized using the GCRMA method [16]. Genes were included for differential expression analysis with a log_2_ GCRMA expression cutoff of at least five GCRMA-normalized expression units. For the Krueger dataset, the BN and DN samples were analyzed on a different date from the CMM samples. The batch effects could confound the comparison between BN/DN and CMM samples. Instead, this study focused on substantiating these genes through their overlap with genes that were identified in the two other independent datasets.

Scatolini and colleagues performed quality control and processing and provided the log_10_ of the red to green signal ratio [12]. Signal was obtained in comparison to a universal human reference (BD™ Human Universal Reference Total RNA, Clontech, Palo Alto, CA, USA). These values were changed to log_2_ for the differential gene expression analysis. Genes were included for differential expression analysis with an absolute log_2_ red to green signal ratio cutoff of at least one. The distribution of the log_2_ GCRMA values was compared to the log_2_ red to green ratio, and a lower expression cutoff was used for the two-channel data given the lower range in expression values compared to the one-channel microarray data.

The RNAseq dataset from Bastian and colleagues was obtained as raw FASTQ files [13]. We used FastQC version 0.11.5 to assess the FASTQ quality (https://www.bioinformatics.babraham.ac.uk/projects/fastqc/). Trimming was performed using TRIMMOMATIC IlluminaClip with the following parameters: seed mismatches 2, palindrome clip threshold 30, simple clip threshold 10, leading quality value 10, trailing quality value 10, sliding window size 4, minimum window quality 15, and minimum read length of 50 [17]. Quality was then re-visualized after trimming (Supplementary Figure S1). Read mapping was performed with Salmon version 0.11.3 [18]. Short sequencing reads from the X and Y chromosomes may mismap due to the high degree of sequence homology. To account for this mismapping, female samples were mapped to the GENCODE GRCh38 reference genome with the Y-chromosome hard-masked, and male samples were mapped to the GRCH38 reference genome with the Y-chromosomal pseudo-autosomal regions hard-masked [19,20]. Raw read counts were summed across all transcripts of the same gene to give a gene-level expression, and genes were only included if they had a known gene-level annotation. Gene expression data were filtered to retain only genes with greater than five reads in at least one sample. The untransformed read counts were then log_10_ counts per million transformed via the voom function [21] from the limma [22] in Bioconductor in R [23] to normalize the variance across expression values.

### 2.2. Differential Expression Analysis

Multidimensional scaling and differential expression were implemented using the limma package for each dataset independently. The final number of genes assessed for each dataset was 16,649 for Krueger, 16,003 for Scatolini, and 19,323 for Bastian. A total of 11,009 genes were shared across all three datasets. The discrepancy in the genes present in the resulting lists is primarily caused by genes that are filtered out by low expression in each dataset. A design matrix was created with lesion type as the predictor value and used to fit the linear model for differential gene expression in each dataset. A linear mixed effects model, which accounts for within-subject correlation, was performed at the gene level using the duplicateCorrelation function for the Bastian dataset. Linear predictors from pairwise comparisons were compared using empirical Bayes statistics with the eBayes function. Genes were considered to be significantly differentially expressed if they had a Benjamini–Hochberg-adjusted *p*-value of less than 0.05, corresponding to a 5% false discovery rate (FDR), and an absolute log_2_ fold change (log_2_(FC)) greater than 1.5. Overlapping gene lists were compiled for genes that were significantly differentially expressed with estimated fold changes in the same direction (e.g., up- or down-regulated) in at least two of the following comparisons: DN vs. CMM (Krueger), DN vs. VGM (Scatolini), or PN vs. CMM (Bastian).

### 2.3. Regularized Regression Analysis

An elastic net, regularized regression model [24,25] was fit for each dataset using the glmnet package in R [26]. Models with greater than two groups were fit using a grouped multinomial regression, whereas those with two groups were fit with a binomial regression. Principal components analysis was performed on the selected genes to allow for visualization as a biplot.

The binomial and multinomial regression models were fit with the assumption of independence between the lesions. One difference in the Bastian binomial regression model is that the PN and CMM included in this dataset are microdissected from a single lesion. While the paired samples are adjusted for in the differential expression analysis, the PN and CMM are treated as two independent groups for the purpose of fitting the regularized regression model. This could introduce a bias into the analysis as it is not directly equivalent to the analysis performed on the Krueger and Scatolini datasets. However, given that the PN and CMM are microdissected from the same lesions, genes that can differentiate these lesions are expected to limit noise and provide a conservative estimate of the changes that occur in melanoma progression.

### 2.4. Assessment of Progression of Genes

Seven genes (CXCL9, CXCL10, FCGR1B, DFNA5, C1QA, PRAME, and SCGB1D2) were selected for further analysis because they were significant (absolute log_2_(FC) greater than or equal to 1.5 and *p*-value less than 0.05) in the Krueger, Bastian, and Scatolini datasets. We visualized the trajectory of expression across lesion types of these seven genes in the Scatolini dataset. The lesion type was presumed to be an ordinal variable where BN = 1, DN = 2, RGM = 3, and VGM = 4. A linear regression was fit between the lesion type and the expression value of each gene independently.

### 2.5. Reactome Over-Representation Analysis

Genes were included in the over-representation analysis if they fit one of the following criteria: (1) selected as one of the top ten differentially expressed genes in a single dataset, (2) selected as a gene in the binomial regression models for each dataset, or (3) selected as an overlapping, significantly differentially expressed gene for any two of the three datasets. This list was analyzed with the Reactome over-representation analysis, which performs a statistical (hypergeometric distribution) analysis to determine if there are more genes in a given pathway than would be expected by chance. Pathways were included in the final pathway diagram if they had a Benjamini–Hochberg-adjusted *p*-value of less than 0.05, corresponding to an FDR of less than 5%.

All of the analyses described here can be reproduced by the code available at: https://github.com/ElizabethBorden/Melanoma_Progression_Gene_Analysis.

## 3. Results

### 3.1. Top Ten Differentially Expressed Genes Differentiate Nevi and Melanoma

Differential expression analysis was performed for each of the pairwise comparisons from the three available datasets. Within the Krueger dataset, 96 genes were significantly differentially expressed between BN and CMM and 37 genes were significantly differentially expressed between DN and CMM (Supplementary Figure S2A). In the Scatolini dataset, 84 genes were significantly differentially expressed between BN and VGM and 83 genes were significantly differentially expressed between DN and VGM (Supplementary Figure S2B). In the Bastian dataset, 1855 genes were differentially expressed between PN and CMM (Supplementary Figure S2C). There were no significantly differentially expressed genes between the BN and DN for Krueger or Scatolini (Supplementary Figure S2A,B). This finding is consistent with the fact that BN and DN are known to be more similar to each other than to CMM. Additionally, the lack of differential expression for the BN vs. DN comparison is consistent across both the Krueger and Scatolini datasets, suggesting that the finding is not a feature of the individual datasets. There were also no significantly differentially expressed genes for DN vs. RGM or BN vs. RGM in the Scatolini dataset (Supplementary Figure S2). A difference in sample collection method may contribute to the greater number of differences between DN and VGM compared to DN and RGM. While the area representative of the lesion was selected from the fresh, excisional biopsy for the BN, DN, and RGM, only the dermal portion was used for the VGM specimen. Complete lists of the differentially expressed genes for every comparison are included in Supplementary Tables S1–S3. Overall, the differential expression analysis highlights the genetic differences that exist between nevi and CMM.

**Figure 1:**
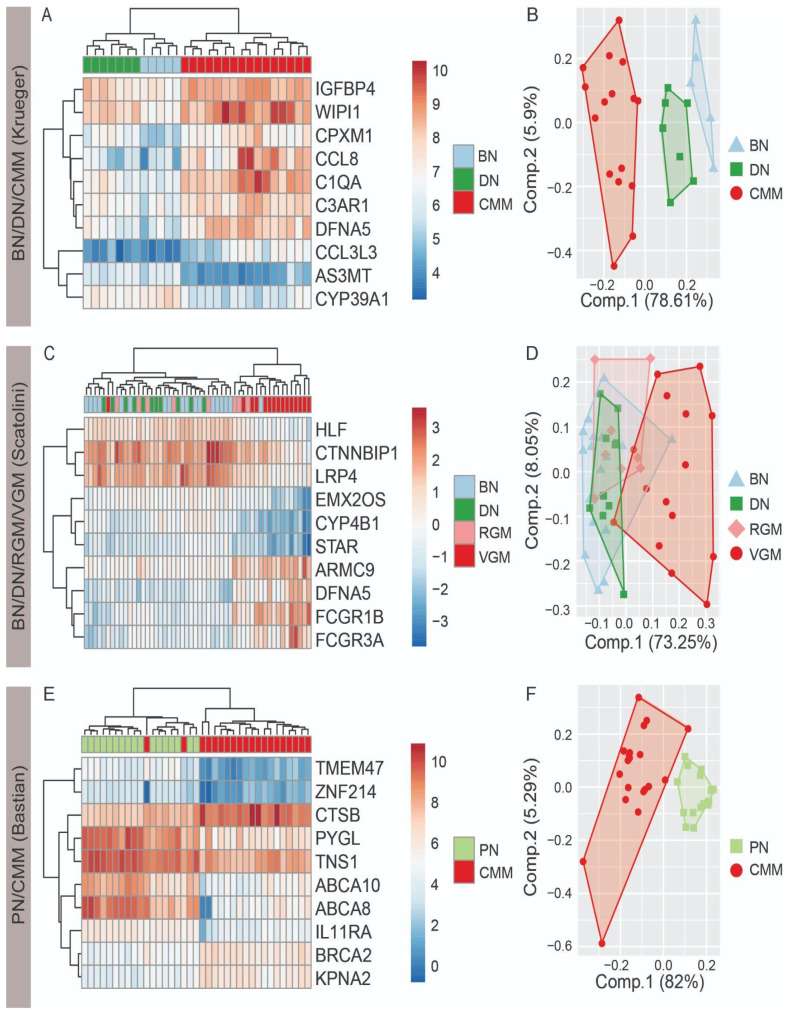
Top ten differentially expressed genes differentiate nevi and melanoma. Differentially expressed genes from each of the pairwise comparisons between the lesions were ranked based on Benjamini–Hochberg-adjusted *p*-values (absolute log_2_(FC) ≥ 1.5; Benjamini–Hochberg-adjusted *p*-value < 0.05; False discovery rate = 5%). Hierarchical clustered heatmap and principal components analysis with the top ten differentially expressed genes for (**A**,**B**) benign nevi (BN), dysplastic nevi (DN), and cutaneous malignant melanoma (CMM) in the Krueger dataset; (**C**,**D**) BN, DN, radial growth phase melanoma (RGM), and vertical growth phase melanoma (VGM) in the Scatolini dataset; and (**E**,**F**) progressing nevi (PN) and CMM for the Bastian dataset. FC, fold change.

To demonstrate whether a small number of genes has the potential to distinguish nevi and melanoma, we tested the ability of the top ten differentially expressed genes to differentiate melanocytic lesions. The top ten differentially expressed genes from any comparison in each dataset were selected based on the lowest Benjamini–Hochberg-adjusted *p*-values and analyzed with both hierarchical clustering and principal components analysis (Figure 1). For the Krueger dataset, there was clear separation between BN, DN, and CMM, with more separation between DN and CMM than BN and DN (Figure 1A,B). For the Scatolini dataset, there was the greatest degree of separation for the VGM, with a large degree of overlap for the BN, DN, and RGM (Figure 1C,D). For the Bastian dataset, there was clear separation between the PN and CMM with the ten selected genes (Figure 1E,F). The gene DFNA5 was found to be upregulated in CMM in the top ten differentially expressed genes for both the Krueger and Scatolini datasets. These findings demonstrate the potential to identify genetic differences between nevi and CMM, and specifically highlight the potential for a small number of genes to accurately differentiate nevi from CMM.

### 3.2. Regularized Regression Models Differentiate Nevi and Melanoma

**Figure 2:**
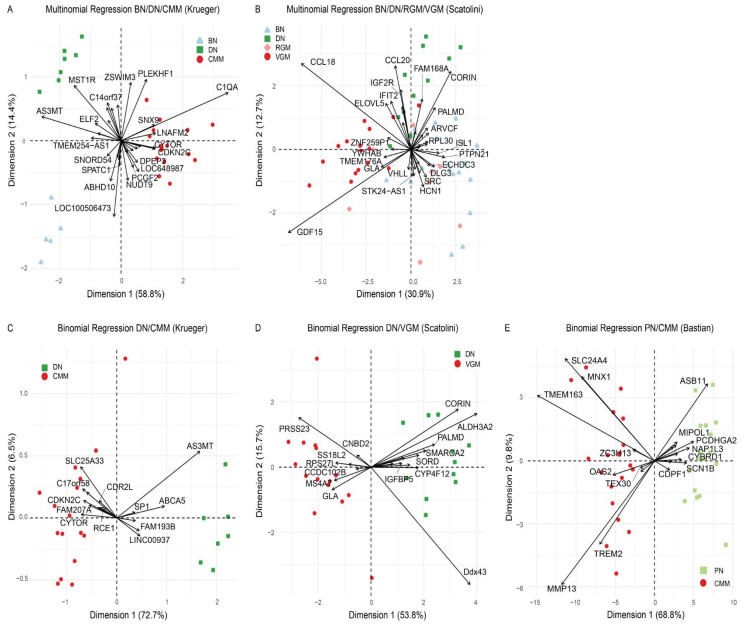
Regularized regression models differentiate nevi and melanoma. Multinomial regression models for (**A**) benign nevi (BN) vs. dysplastic nevi (DN) vs. cutaneous malignant melanoma (CMM) in the Krueger dataset and (**B**) BN vs. DN vs. radial growth phase melanoma (RGM) vs. vertical growth phase melanoma (VGM) in the Scatolini dataset. Binomial regression models for (**C**) DN vs. CMM in the Krueger dataset, (**D**) DN vs. VGM in the Scatolini dataset, and (**E**) progressing nevi (PN) vs. CMM in the Bastian dataset.

To isolate the genes best able to predict the different lesion types in melanoma progression, regularized regression models were applied. Multinomial regularized regression models were used to study the comparison of BN, DN, and CMM in the Krueger dataset (Figure 2A, Supplementary Table S4) and the comparison of BN, DN, RGM, and VGM in the Scatolini dataset (Figure 2B, Supplementary Table S5). In the Krueger dataset, the multinomial regularized regression model was able to classify BN, DN, and CMM lesions. In the Scatolini dataset, the regularized regression differentiated DN, and VGM well, but did not show much differentiation between the BN, DN, and RGM. To focus the modeling on identifying genes that predict progression from nevi to melanoma, binomial regression models for DN or PN and melanoma were fit for all three datasets. VGM samples were used for the Scatolini dataset since the RGM included Clark level I samples, which are melanoma in situ, and Clark level II samples, which are microinvasive melanoma, so VGM was thought to be more similar to the CMM from the other datasets. Additionally, there were no significantly differentially expressed genes between DN and RGM. All three datasets showed separation of the nevi and melanoma (DN vs. CMM for Krueger, DN vs. VGM for Scatolini, and PN vs. CMM for Bastian) based on a small number of genes (Figure 2C–E, Supplementary Tables S6–S8). There were no overlapping genes selected in the binomial and multinomial regression models. These findings demonstrate that a small number of genes selected by regularized regression models differentiate nevi and melanoma.

### 3.3. Differentially Expressed Genes between Stages of Melanoma Progression Overlap between Multiple Datasets

**Figure 3:**
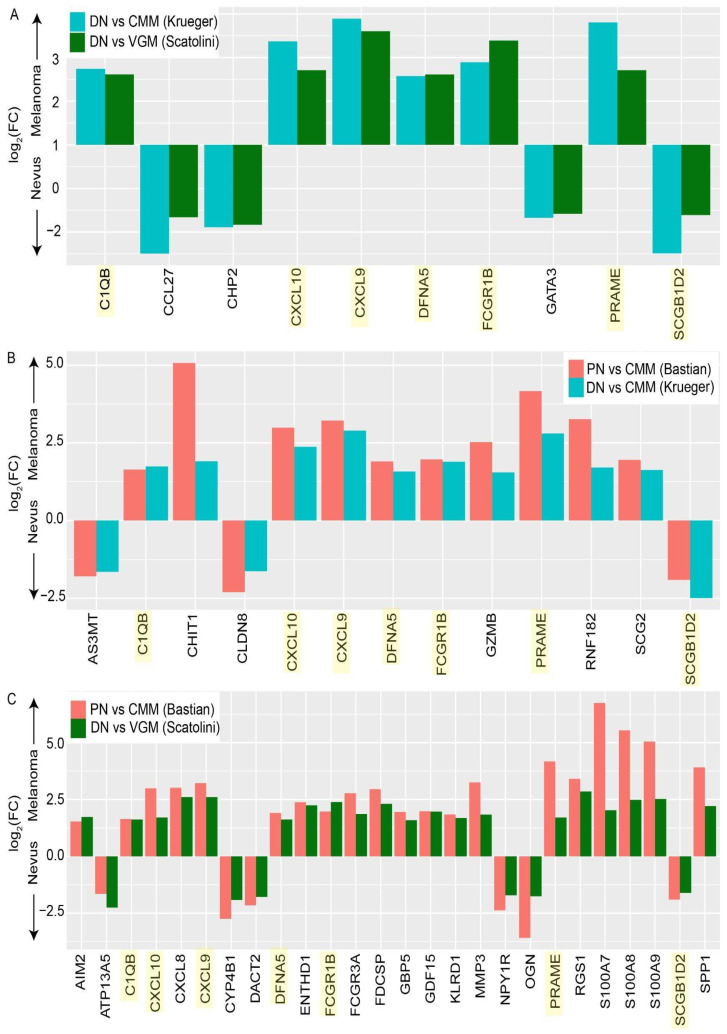
Differentially expressed genes between nevi and melanoma overlap between multiple datasets. Overlapping, significantly differentially expressed genes between (**A**) dysplastic nevi (DN) vs. cutaneous malignant melanoma (CMM) in the Krueger dataset and DN vs. vertical growth phase melanoma (VGM) in the Scatolini dataset, (**B**) DN vs. CMM in the Krueger dataset and progressing nevi (PN) vs. CMM in the Bastian dataset, and (**C**) DN vs. VGM in the Scatolini dataset and PN vs. CMM in the Bastian dataset. The bars represent the log_2_(FC) of the gene in each dataset. Genes with a log_2_(FC) above zero are upregulated in melanoma, and genes with a log_2_(FC) below zero are upregulated in nevi. Genes were overlapping if they had an absolute log_2_(FC) ≥ 1.5, a Benjamini–Hochberg-adjusted *p*-value < 0.05, and the log_2_(FC) was in the same direction for both datasets. Genes that were significant across all three datasets are highlighted in yellow. FC, fold change.

To identify genes with the potential to distinguish DN or PN from CMM, we isolated those genes that were significantly differentially expressed in a consistent direction across the DN or PN to CMM comparisons in multiple datasets. There were ten genes in common between the DN vs. CMM from the Krueger dataset and the DN vs. VGM from the Scatolini dataset (Figure 3A), 13 genes in common between the DN vs. CMM from the Krueger dataset and the PN vs. CMM from the Bastian dataset (Figure 3B), and 26 overlapping genes between the DN vs. VGM in the Scatolini dataset and the PN vs. CMM in the Bastian dataset (Figure 3C). Seven genes were found to be in common across all three datasets—CXCL9, CXCL10, FCGR1B, DFNA5, C1QB, PRAME, and SCGB1D2. These are genes of particular interest for a gene signature of progression from nevi to CMM, given that they are consistent across all three datasets.

**Figure 4:**
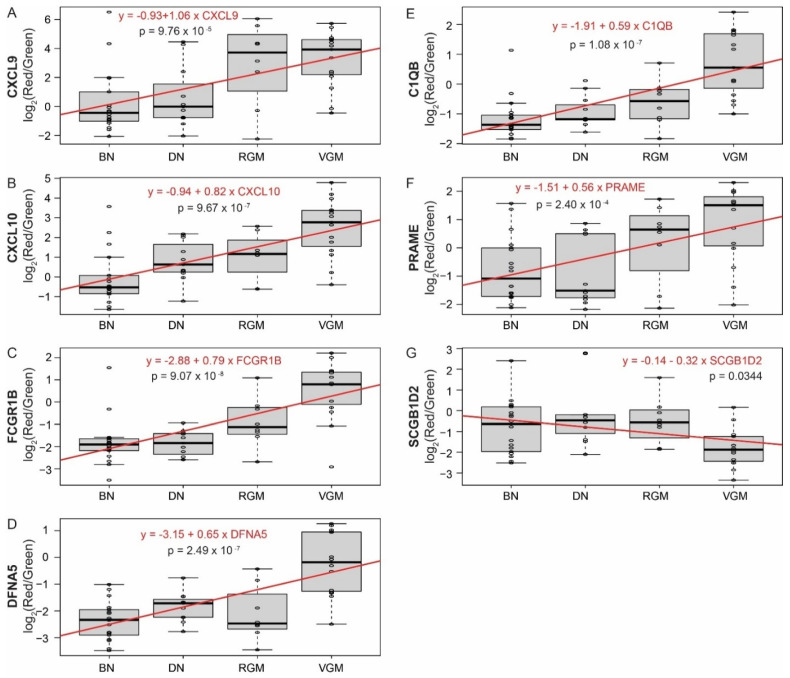
Linear change in expression of CXCL9, CXCL10, FCGR1B, DFNA5, C1QB, PRAME, and SCGB1D2 across lesions in the spectrum of melanoma progression. Linear regression between lesion type and indicated gene expression in the Scatolini dataset. (**A**) CXCL9, (**B**) CXCL10, (**C**) FCGR1B, (**D**) DFNA5, (**E**) C1QB, (**F**) PRAME, and (**G**) SCGB1D2. The bold line in the box plot indicates the median; the upper and lower limits of the boxes indicate the 75th and 25th percentiles, respectively. The lower and upper whiskers indicate the minimum and maximum. Dots outside of the box and whiskers indicate outliers. The red line demonstrates the linear model fit between lesion type and gene expression; the equation of the linear regression is included in the red text. *p*-values indicate the significance of the association between the lesion type and expression of the gene of interest. BN, benign nevi; DN, dysplastic nevi; RGM, radial growth phase melanoma; VGM, vertical growth phase melanoma.

To further substantiate the association of these genes with melanoma progression, the expression of each of the genes identified across the three datasets was compared to the lesion types from the Scatolini dataset, since the Scatolini dataset had the greatest number of lesion types between BN and invasive melanoma (BN, DN, RGM, and VGM). All seven genes tested showed a significant correlation with the progression from BN to DN to RGM to VGM (Figure 4A–E). Melanomagenesis is explained through a linear model under the assumption that the relationship between each pair of lesion subtypes is the same, resulting in a single coefficient that describes the increasing trajectory in progression. CXCL9 showed the greatest degree of change across the lesion types with a linear regression coefficient of 1.06. CXCL10, FCGR1B, DFNA5, C1QB, and PRAME also showed positive associations with progression, with coefficients of 0.82, 0.79, 0.65, 0.59, and 0.56, respectively. SCGB1D2 showed a decrease in expression with melanoma progression with a coefficient of −0.32, consistent with a prior study [27]. The change in expression of these genes among lesions in the spectrum of melanoma progression provides additional evidence that these genes are strong candidates for indicating melanoma progression.

### 3.4. Genes Associated with Melanoma Progression Show Significant Enrichment of Multiple Pathways Related to the Immune System

**Figure 5:**
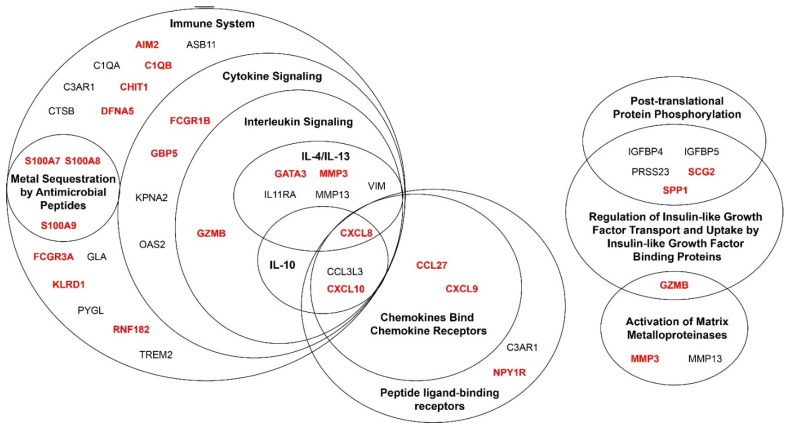
Genes associated with melanoma progression show significant enrichment of multiple pathways related to the immune system. Pathway enrichment was performed using Reactome’s over-representation analysis on a combination of genes identified by the following analyses: (1) top ten differentially expressed gene in melanoma progression in at least one dataset (Figure 1); (2) gene identified in at least one binomial regression model distinguishing nevi vs. cutaneous malignant melanoma (CMM) (Figure 2C–E); and (3) a significantly differentially expressed gene between nevi and CMM in at least two datasets (Figure 3). Genes colored in red and bolded represent genes that were differentially expressed between nevi and CMM in at least two datasets (Figure 3). A pathway was considered to be enriched if it had a Benjamini–Hochberg-adjusted *p*-value < 0.05 (corresponding to a false discovery rate < 5%). The eleven significantly enriched pathways identified are each represented as a circle, with nested circles indicating pathways that are subsets of one another. IL-10, interleukin-10, IL-4, interleukin-4, IL-13, interleukin-13.

To assess the broader biological implications of the genes associated with melanoma progression from each analysis, over-representation analysis was performed with Reactome to identify the pathways over-represented or enriched in this gene set (Figure 5). All genes included in the Reactome analysis are listed in Supplementary Table S9. There was significant enrichment of genes in multiple immune-related pathways, including chemokine receptors bind chemokines, interleukin (IL)-10 signaling, IL-4 and IL-13 signaling, metal sequestration by antimicrobial proteins, cytokine signaling in immune system, signaling by interleukins, and immune system. Smaller sets of genes were related to the regulation of insulin-like growth factor transport and uptake by insulin-like growth factor binding proteins (IGFBP), post-translational protein phosphorylation, and activation of matrix metalloproteinases. Five out of the seven significantly differentially expressed genes in all three datasets fell under one of these significantly enriched pathways. The two genes that did not, PRAME and SCGB1D2, are not annotated in the Reactome database. The Reactome enrichment analysis was also performed with a more conservative set of genes that included only those genes that were significantly differentially expressed in at least two datasets. All of the pathways identified with the full set were also significantly enriched in the reduced set except for post-translational protein phosphorylation. Overall, these results indicate that, in addition to overlapping genes selected across the datasets, there are overlapping pathways that are modulated in melanomagenesis, most notably, immune-related pathways.

## 4. Discussion

There is a paucity of studies investigating the gene signatures associated with the progression from DN and PN to CMM. Differential gene expression analysis identified shared changes in gene expression and pathways from nevi to CMM across three datasets. Seven genes were consistently, differentially expressed between DN or PN and CMM across each of the three datasets: C1QB, CXCL9, CXCL10, DFNA5, FCGR1B, PRAME, and SCGB1D2. In addition to having consistent up or downregulation from nevi to CMM, these genes demonstrated linear change from BN to DN to RGM to VGM. Thus, these genes represent the top candidates for a gene signature indicating progression from nevi to CMM, based on our analyses.

The significantly, differentially expressed genes and genes selected in binomial regression models between nevi and CMM demonstrate a striking enrichment in immune system-related pathways. Given the immune system’s ability to sense dangerous changes within the body, the immune system is well positioned to detect and react to the progression from nevus to CMM. Thus, the enrichment in immune-related pathways distinguishing nevi from CMM is not surprising. Given possible opposing functions of the same gene depending on which cell type it is expressed in, complexities of each pathway, and the interplay of numerous pathways, it is not possible to extrapolate whether these changes in gene expression result in an overall immune-activating or immunosuppressive environment. It is also important to note that many of these genes also have functions outside of the immune system and the cell of origin is not known in the bulk RNAseq or microarray data. Our study demonstrates an association of changes in immune-related pathways with the progression from nevi to CMM. Many of the enriched pathways are related to chemokines and cytokines, which are responsible for cell signaling and communication in the immune system.

Many genes in the significantly enriched pathways have been previously associated with cancer. A review of prior literature on these genes is provided in Table 2, where studies in melanoma were prioritized and studies in other cancers were included where studies in melanoma were unavailable. The genes from the pathway analysis can be broadly divided into immune- and non-immune-related genes. Within the immune-related genes, enriched pathways were identified for chemokines, peptide ligand-binding receptors, various interleukin signaling pathways, cytokines, and metal sequestering antimicrobial peptides. Within the cluster for chemokines and peptide ligand-binding receptors, CXCL8, CXCL9, and CXCL10 have been previously shown to have increased expression in CMM compared to nevi, in agreement with our study [28,29,30]. CXCL8, CXCL9, CXCL10, CCL27, and C3AR1 have known immunomodulatory roles in melanoma [31,32,33,34,35,36]. In the interleukin-4 and interleukin-13 signaling pathway, CXCL8 and MMP3 have published roles in melanoma growth and metastasis [33,34,35,37]. Within the cytokine signaling pathway, GBP5 and KPNA2 have known roles in the immunomodulation and progression of melanoma, respectively [38,39,40,41]. Additionally, GZMB-expressing cells are increased in DN (severe atypia) and CMM compared with BN and DN (mild atypia) [42]. Within the metal sequestration by the antimicrobial peptides pathway, the S100 family genes S100A7, S100A8, and S100A9 have been linked to tumor growth and metastasis in multiple cancers, including melanoma [43,44]. These genes are distinct from S100B, which is used in the diagnosis of melanoma by immunohistochemistry. Within the immune system broadly, a large number of genes were identified with known roles in cancer, including AIM2 [45], C1QA [46], and DFNA5 (also known as GSDME) [47], which have been identified to have a mechanistic role in melanoma tumor growth. Additionally, our study identified genes without reported expression in CMM and nevi or biological function related to melanoma progression, including CCL3L3, NPY1R, IL11A, FCGR1B, OAS2, ASB11, FCGR3A, and GLA. Overall, our study has identified many immune genes that are known to be associated with melanoma progression and new genes of interest that are likely associated with melanoma progression.

In addition to the immune pathways that show enrichment during the progression of melanoma, clusters of genes were also identified for post-translational phosphorylation, regulation of insulin-like growth factor transport, and activation of matrix metalloproteinases. The genes in matrix metalloproteinases are also contained in immune-related pathways and are not separated out here. Genes in the regulation of the insulin-like growth factor transport pathway have known functions in cancer proliferation and migration, including IGFBP5 [48], PRSS23 [49,50], SCG2 [51,52], and SPP1 [53,54]. The enriched genes in the non-immune pathways have roles in cell proliferation and migration, and the differential gene expression observed in CMM is consistent with their known function.

**Table 2:** Summary of the reported expression and function of the genes identified in the pathway analysis.

Gene	Datasets *			Direction	Reported Expression and Function
	K	S	B		
**Chemokines Bind Chemokine Receptors**					
**CXCL8**				↑CMM	Protein expression increased in CMM vs. nevi by IHC [28,29]
					Promotes melanoma progression and metastasis [33,34,35]
**CXCL9** **CXCL10**				↑CMM	CXCL9 and CXCL10 increased gene expression in CMM vs. BN by qRT-PCR [30]
					CXCL9 and CXCL10 in gene signature that differentiates BN and CMM [55,56,57]
					CXCL9 increased gene expression in melanoma metastases vs. BN [58]
					CXCL9 and CXCL10 recruits CXCR3-expressing effector T cells and natural killer cells into melanoma [59]
					CXCL10 binds CXCR3 on tumor cells to promote metastases [36]
**CCL27**				↓CMM	Recruits T cells in melanoma mouse model [32]
**Peptide Ligand-Binding Receptors**					
**C3AR1**				↑CMM	Increases melanoma tumor growth by inhibiting neutrophil and CD4+ T cell responses [31]
**IL-4 and IL-13 Signaling Pathways**					
**GATA3**				↓CMM	Stabilizes HIF-1α to enhance cancer invasiveness under hypoxic conditions [60]
**MMP3**				↑CMM	Protein expressed in CMM lesions, but not normal skin, by IHC and IF [61]
					Promotes melanoma tumor growth and metastasis [37]
**MMP13**				↑CMM	Protein expression in some CMM cases, absent in BN, by IHC [62]
**VIM**				↓CMM	Protein uniformly expressed in BN and CMM melanocytes by IF [63]
**Cytokine Signaling**					
**GZMB**				↑CMM	Increased GZMB-expressing cells in DN (severe atypia) and CMM vs. BN and DN (mild atypia) by IHC [42]
**FCGR1B**				↑CMM	Induced by interferon-γ [64,65]
**GBP5**				↑CMM	Induced by interferon-γ and type 1 interferons
					Stimulates assembly of NLRP3 inflammasome [39], which expands myeloid-derived suppressor cells in melanoma leading to immunosuppression and increased tumor growth [40]
**KPNA2**				↑CMM	Promotes proliferation, migration, and invasion in melanoma cells [38,41]
**OAS2**				↑CMM	Induced by type 1 interferons and important in anti-viral immune response
					Gene expression induced by UVB in human primary melanocytes (qRT-PCR) [66]
**Metal Sequestration by Antimicrobial Peptides**					
**S100A7** **S100A8** **S100A9**				↑CMM	S100A7, S100A8, and S100A9 increased gene expression in CMM vs. BN by qRT-PCR [55,56,57]
					S100A7, S100A8, and S100A9 in gene signature that differentiates BN and CMM [55,56,57]
					S100A7, S100A8, and S100A9 linked to tumor growth and metastasis in multiple cancers [43]
					S100A9 promotes migration and metastasis of EMMPRIN-expressing melanoma cells [44]
**Immune System**					
**AIM2**				↑CMM	Expression in dendritic cells promotes immunosuppressive tumor microenvironment and increased melanoma tumor growth [45]
**ASB11**				↓CMM	Downregulated by DNA damage, stabilizes pro-apoptotic protein BIK, which increases apoptosis [67]
**C1QA**				↑CMM	C1q protein (composed of C1QA, C1QB, and C1QC) expressed by mesenchymal cells and immune cells in melanoma by IHC [46] C1qa-/- mice have slower melanoma tumor growth, prolonged survival, decreased tumor angiogenesis, and decreased lung metastasis [46] C1q from non-bone marrow-derived cells promotes accelerated melanoma tumor growth [46] C1q promotes cell adhesion, migration, and proliferation of melanoma cells [46]
**C1QB**				↑CMM	
**DFNA5/** **GSDME**				↑CMM	Gene expression increased in CMM vs. normal skin by RNAseq [68]
					Included in pyroptosis-related gene signature for BN vs. CMM [68]
					Triggers pyroptosis and apoptosis [47]
					Deficiency in melanoma cells increases in vitro and in vivo tumor growth [47]
**CHIT1**				↑CMM	Produced by macrophages stimulated by interferon-*γ* and tumor necrosis factor-*α* [69]
					Increases transforming growth factor-*β* SMAD signaling [69], which plays role in melanoma metastasis [70] and angiogenesis in various cancers [71]
**CTSB**				↑CMM	Involved in metastasis [72,73], angiogenesis, and invasion of tumor cells, including melanoma [74,75,76]
**FCGR3A**				↑CMM	Expressed on subset of natural killer cells [77] Mediates antibody-dependent cell-mediated cytotoxicity [77]
**GLA**				↓CMM	Patients with GLA deficiency possibly have increased rate of melanoma [78]
**KLRD1**				↑CMM	Protein expressed on tumor infiltrating lymphocytes in CMM by IHC [79]
					Regulates natural killer cell cytotoxicity [79]
**PYGL**				↓CMM	Differentially expressed in BN with and without V600E BRAF mutation by microarray [80]
					Involved in glycogen metabolism, which regulates inflammatory responses and tumorigenesis [81,82,83]
**RNF182**				↑CMM	Suppresses Toll-like receptor-triggered immune response and decreases production of proinflammatory cytokines [84]
**TREM2**				↑CMM	Encodes for innate immune receptor on tumor infiltrating myeloid cells [85]
					TREM2 deletion decreases immunosuppressive regulatory myeloid cells within tumors, which decreases exhausted CD8+ T cell subsets and tumor growth [86]
**Regulation of Insulin-like Growth Factor Transport and Uptake by Insulin-like Growth Factor Binding Proteins**					
**IGFBP4**				↑CMM	Protein expressed in CMM by IHC [87]
**IGFBP5**				↓CMM	Gene and protein expression increased in CMM vs. nevi by qRT-PCR and IHC [48]
					Gene expression increased in metastatic melanoma vs. BN by RNAseq [58]
					Overexpression inhibits in vitro proliferation, migration, invasion, epithelial to mesenchymal transition, and in vivo tumor growth and metastasis of melanoma cell lines [48]
**PRSS23**				↑CMM	Knockdown decreases cancer cell proliferation in breast and gastric cancer [49,50]
**SCG2**				↑CMM	Gene expression increased in VGM and metastatic melanoma vs. normal skin, BN, and melanoma in situ by microarray [27]
					Increases migration of melanoma cells [52]
					Plays role in chemoattraction and migration [51]
**SPP1**				↑CMM	Increased expression in CMM vs. BN across 5 studies (RNAseq, microarray, IHC) [58,88,89,90,91]
					Included in 5 protein assay that distinguishes BN vs. CMM by IHC [89]
					SPP1 treatment increases melanoma cell migration, invasion, and proliferation [54]
					SPP1 knockdown decreased in vitro and in vivo proliferation, migration, and invasion of melanoma cells [53]

* K, Krueger; S, Scatolini; B, Bastian; IF, immunofluorescence; IHC, immunohistochemistry; qRT-PCR, quantitative reverse transcription polymerase chain reaction; RNAseq, RNA sequencing.

Clinical tests that distinguish nevi from CMM include genes identified in our analyses: PRAME, CXCL9, CXCL10, S100A7, S100A8, and S100A9. While PRAME was not identified in the pathway analysis, because PRAME is not annotated in the Reactome database, we found that PRAME RNA expression was increased in CMM compared to nevi in each of the three datasets. PReferentially expressed Antigen in MElanoma (PRAME) expression is known to be increased in melanoma compared to nevi by microarray, RT-PCR, and immunohistochemistry [11,88,92]. Our findings are consistent with a prior study identifying PRAME protein expression in a portion of melanocytes in a minority of BN and DN and diffuse expression in melanoma cells in CMM [92]. In support of the utility of PRAME gene expression as a biomarker of melanoma progression, the Pigmented Lesion Assay uses the expression of two genes (PRAME and LINC00518) to differentiate clinically atypical nevi from CMM [93,94]. Similarly, we found LINC00518 expression was significantly higher for CMM than BN and DN in the Krueger dataset and significantly higher for VGM compared to RGM in the Scatolini dataset. The myPath Melanoma Test utilizes the expression of 14 signature genes, including PRAME, CXCL9, CXCL10, S100A7, S100A8, and S100A9, and nine reference genes to distinguish BN from CMM [55,56,57]. Thus, the association of PRAME, CXCL9, CXCL10, S100A7, S100A8, and S100A9 with melanoma progression is supported by prior studies and commercially available clinical tests.

This study successfully demonstrated a set of seven genes that are candidates for a gene signature for melanoma progression. Not only did these seven genes show consistency across three independent datasets, but their expression also showed a linear, correlative relationship with melanomagenesis through progression from BN to DN to RGM to VGM subtypes. Despite these successes, the primary limitation of this study was the inability to generate a gene signature biomarker for progression across datasets. Methods of biomarker development can be differentiated from hypothesis testing (i.e., differentially expressed genes) in both variable selection (genes selected) and variable reduction (linear combination); methods of biomarker development do not use test statistics or *p*-values. As previously shown, there is no unique (one-to-one) correspondence (mathematically) between estimates used in hypothesis testing (e.g., means, fold-change, odds ratios) and measures of prediction (sensitivity, specificity) [95]. Because of the differences in hypothesis testing and biomarker development, the seven differentially expressed genes do not represent a gene signature biomarker. There are multiple contributory factors that might have impacted the ability to generate a gene signature biomarker, the most obvious of which are the small sample sizes and different technologies used to evaluate gene expression. The impact of different technologies can be lessened by standardizing data either in a similar fashion (as carried out in this manuscript) or standardizing across studies, which is only possible when study inclusion/exclusion and sample process are equivalent across studies. Other known considerations for the development of biomarker signatures include other REMARK criteria [96], including patient-level characteristics; prior treatments; and study design differences. Another limitation of this study includes that CMM subtype and thickness were not available in the datasets used, and thus, were not accounted for in this analysis.

A potentially successful approach for the development of a melanoma progression biomarker signature, from a design perspective, would be retrospective use of a completed clinical trial or convenience sample with a follow-on measure of features/genes utilizing a prospective-specimen-collection, retrospective-blinded-evaluation (PRoBE) design [95]. This approach can be tied to a level-of-evidence described by Simon et.al. [97] when using archived samples for this purpose; success in accurately predicting progression from prospective sample collection is the highest level of evidence afforded prognostic and predictive biomarkers. Biomarker signatures are generally summarized using linear or non-linear combinations of the chosen features/genes that reduce the dimensionality and create a single qualitative or quantitative score. These signatures can include a combination of clinical and molecular features or the molecular features alone, i.e., the Oncotype Dx Recurrence Score^®^ Test. In order to be approved in a clinical setting, these scores are necessarily qualitative to remove subjectivity of their application. Performance metrics for biomarker signatures are typically based on predictive capacity (AUC, Brier score, misclassification rate) in an independent study sample, often called the “test sample” after being developed (or “trained”) in an independent set of samples. The accuracy of a prognostic signature, in our case one that describes melanoma progression, will ultimately need to be performed prospectively with the goal of clinical utility. Clinical utility can be demonstrated by classification accuracy, e.g., accurately identify “at risk” individuals with melanoma progression. Additionally, statistical decision theory allows for simultaneous evaluation of statistical predictive performance of the biomarker signature as well as clinical usefulness, described by patient-level benefit of early detection [97].

Given the success and limitations of the analysis of the publicly available data, the best practices for early biomarker development should include evaluation of the optimal criteria for sample procurement. For example, prospective sample procurement should limit nuisance variation (variation that is not of clinical interest), by restricting sample procurement based on the clinical need to identify a biomarker that distinguishes nevi from early CMM. Two considerations include CMM subtype and thickness. The association of superficial spreading melanoma with DN is higher than other CMM subtypes, with 18–32% of superficial spreading melanomas arising in the context of DN [98,99,100]. Patients with DN are more likely to have a prior diagnosis of superficial spreading melanoma compared with nodular melanoma [101]. In addition, the association of CMM with DN is higher in thin CMM [100,101], which represents early disease. Analysis of the seven genes that were differentially expressed between nevi and CMM revealed consistent directionality between BN and thin CMM (<0.8 mm Breslow depth) in an independent dataset from Kunz and colleagues [102]; although, one gene, SCGB1D2, was no longer statistically significant due to high variance (data not shown). These results support that these genes may be differentially expressed between nevi and early CMM. To generate a gene signature, future prospective studies should focus on the comparison between DN and thin, superficial spreading CMM.

## 5. Conclusions

Given the need to identify a gene signature biomarker of melanoma progression, we undertook differential gene expression analysis and regularized regression modeling of three publicly available datasets to identify genes associated with the progression from nevi to CMM. In each of the three datasets, the expression of six genes, C1QB, CXCL9, CXCL10, DFNA5, FCGR1B, and PRAME, was increased in CMM compared to DN or PN, and the expression of SCGB1D2 was decreased in CMM compared to DN or PN. The genes identified that distinguish nevi and CMM show significant enrichment in multiple immune system-related pathways. The biological significance of many of the genes identified in this study is supported by prior studies demonstrating increased expression in melanoma and/or functions consistent with melanoma progression. This study demonstrates (1) a novel application of bioinformatic approaches to aid clinical trials of melanoma prevention strategies and (2) the feasibility of determining a gene signature biomarker of melanoma progression in a clinically annotated convenience or retrospectively selected set of samples, with a prospective study follow-on for clinical utility evaluation.

## Figures and Tables

**Table 1:** Three available datasets with dysplastic or progressing nevi and melanoma.

Lesion Types ^1^ (n)	Sample Type	Relationship	Data Type	Data Access	References
BN (5), DN (7), CMM (16)	Frozen sections	Independent samples	1-channel microarray	GSE114445	**Krueger:**
					Mitsui et al., 2016 [10]
					Yan et al., 2019 [11]
BN (18), DN (11), RGM (8), VGM (15)	Fresh biopsy (VGM limited to dermal portion)	Independent samples	2-channel microarray	GSE12391	**Scatolini** et al. 2010 [12]
PN (17), CMM (20)	Manual microdissection of FFPE sections	Paired lesions	RNAseq	phs001550.v2.pl	**Bastian:**
					Shain et al., 2015 [13]
					Shain et al., 2018 [14]

^1^ Benign nevi (BN), dysplastic nevi (DN), cutaneous malignant melanoma (CMM), progressing nevi (PN), radial growth phase melanoma (RGM), vertical growth phase melanoma (VGM).

## Data Availability

Publicly available data were used for this study. The data from Shain et al. (Bastian) presented in this study are available in dbGaP with the following accession number: phs001550.v2.p1. The data from Yan et al. (Krueger) presented in this study are available from GEO with accession number GSE114445. Finally, the data from Scatolini et al. are available from GEO with accession number GSE12391. All of the analysis described here can be reproduced by the code available at: https://github.com/ElizabethBorden/Melanoma_Progression_Gene_Analysis.

## Supplementary Materials

The following are available online at https://www.mdpi.com/article/10.3390/cancers14010003/s1, Figure S1: Mean quality scores (phred scores) across each base pair position for each of the samples from the Bastian dataset, Figure S2: Differentially expressed genes suggest potential to distinguish melanoma from nevi, Table S1: Differentially expressed genes in the Krueger dataset, Table S2: Differentially expressed genes in the Scatolini dataset, Table S3: Differentially expressed genes in the Bastian dataset, Table S4: Genes and coefficients for the Krueger multinomial regression model, Table S5: Genes and coefficients for the Scatolini multinomial regression model, Table S6: Genes and coefficients for the Krueger binomial regression model, Table S7: Genes and coefficients for the Scatolini binomial regression model, Table S8: Genes and coefficients for the Bastian binomial regression model, Table S9: All genes considered for the Reactome analysis (Figure 5).
