# Supplementary material for: Shared Gene Expression and Immune Pathway Changes Associated with Progression from Nevi to Melanoma
Source: Cancers (Basel). 2021 Dec 21;14(1):3. doi: 10.3390/cancers14010003 (PMC8749980; doi:10.3390/cancers14010003)
Supplement: Supplementary file 1 [file cancers-14-00003-s001.zip › supplementary figures.pdf]

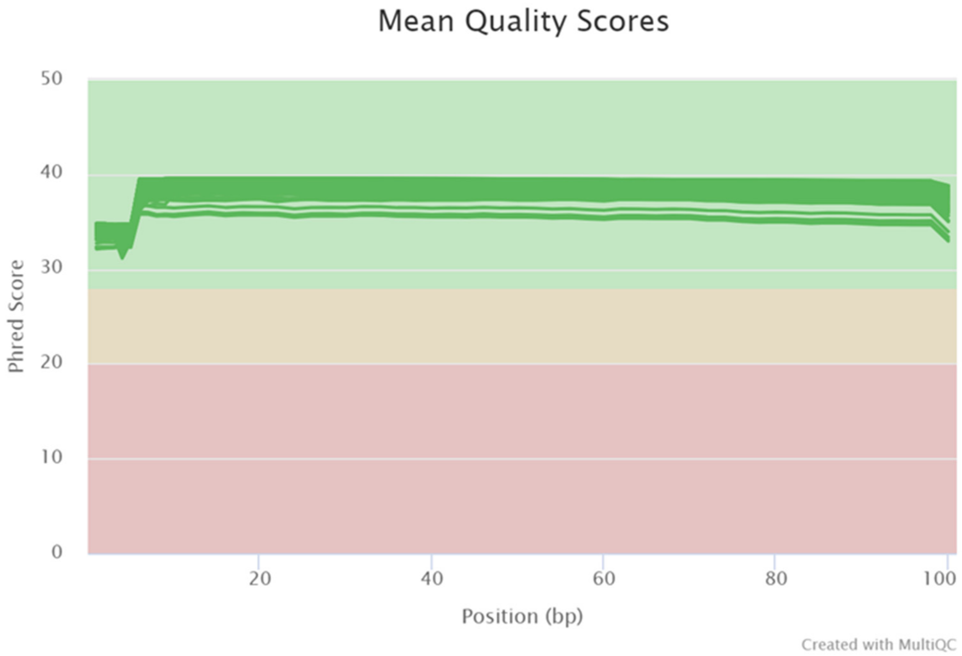


**Supplementary Figure S1.** Mean quality scores (phred scores) across each base pair position for each of the samples from the Bastian dataset. Default phred thresholds from FastQC were used to define the color blocks. Green indicates high quality calls (phred score above 28), yellow indicates reasonable quality calls (phred score between 20 and 28) and red indicates poor calls (phred score below 20).

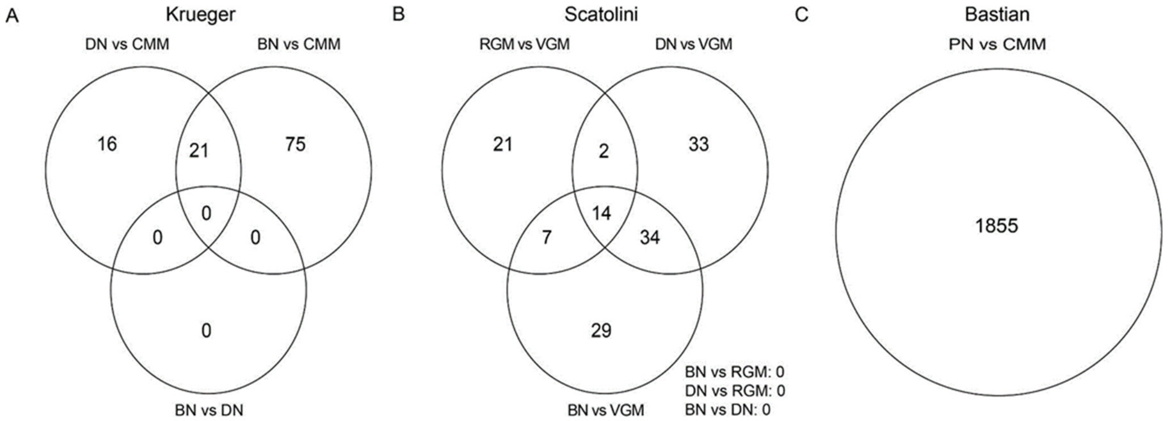


**Supplementary Figure S2.** Differentially expressed genes suggest potential to distinguish melanoma from nevi. Significantly differentially expressed genes (absolute log2(FC)) ≥ 1.5; Benjamini-Hochberg adjusted p-value < 0.05; False discovery rate = 5%) for each pairwise comparison from the (A) Krueger dataset, (B) Scatolini dataset, and (C) Bastian dataset. The comparisons for the Scatolini dataset of BN vs. RGM, DN vs. RGM, and BN vs. DN all contained zero differentially expressed genes. BN, benign nevi; CMM, cutaneous malignant melanoma; DN, dysplastic nevi; FC, fold change; RGM, radial growth phase melanoma; VGM, vertical growth phase melanoma.
